# Synthesis, Characterization of Liposomes Modified with Biosurfactant MEL-A Loading Betulinic Acid and Its Anticancer Effect in HepG2 Cell

**DOI:** 10.3390/molecules24213939

**Published:** 2019-10-31

**Authors:** Qin Shu, Jianan Wu, Qihe Chen

**Affiliations:** Department of Food Science and Nutrition, Zhejiang University, Hangzhou 310058, China; 21713041@zju.edu.cn (Q.S.); 21613036@zju.edu.cn (J.W.)

**Keywords:** Liposomes, betulinic acid (BA), mannosylerythritol lipid-A (MEL-A), HepG2, anticancer activity

## Abstract

As a novel natural compound delivery system, liposomes are capable of incorporating lipophilic bioactive compounds with enhanced compound solubility, stability and bioavailability, and have been successfully translated into real-time clinical applications. To construct the soy phosphatidylcholine (SPC)–cholesterol (Chol) liposome system, the optimal formulation was investigated as 3:1 of SPC to Chol, 10% mannosylerythritol lipid-A (MEL-A) and 1% betulinic acid. Results show that liposomes with or without betulinic acid or MEL-A are able to inhibit the proliferation of HepG2 cells with a dose-effect relation remarkably. In addition, the modification of MEL-A in liposomes can significantly promote cell apoptosis and strengthen the destruction of mitochondrial membrane potential in HepG2 cells. Liposomes containing MEL-A and betulinic acid have exhibited excellent anticancer activity, which provide factual basis for the development of MEL-A in the anti-cancer applications. These results provide a design thought to develop delivery liposome systems carrying betulinic acid with enhanced functional and pharmaceutical attributes.

## 1. Introduction

Great attention was paid to the study of pharmacokinetics and the development of biopharmaceutics in the second half of the last century, when enormous progress was obtained in the field of pharmaceutical industry [1]. With the thought of a controlled and targeted drug delivery system first coming out in the world, nanotechnology has become more involved in the drug field in the form of submicron nanoparticles [1]. Liposomes, described as spherical lipid vesicles with phospholipid bilayer structure [2], were one of the first nano-sized drug delivery systems, which has been successfully produced and applied into clinical fields. After discovered in 1961 by Alec Bangham et al. [3], lots of researchers have declared great applications of liposomes in diverse fields, such as delivery of drugs, biomolecules and genes [4]. Due to the extensive development of liposome technology, plenty of drug carriers based on liposomes are available for improving the treatment of disease in pharmaceutical industry. Actually, encapsulating drugs in liposomes has advantage in enhancing therapeutic index by means of changes in their pharmacokinetics and pharmacodynamics [5]. As for drugs with different solubility, there are different ways to be encapsulated in liposomes: hydrophobic compounds are embedded in the phospholipid bilayer, and hydrophilic compounds are entrapped in the aqueous cavity.

**Figure 1:**
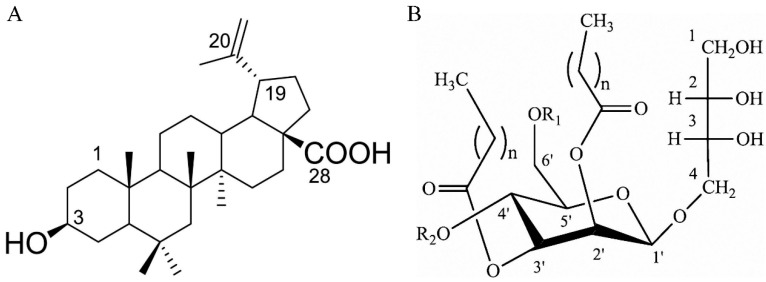
(**A**) The structure of betulinic acid (BA). (**B**) The structure of mannosylerythritol lipids (MELs). MEL-A: R_1_ = R_2_ = Ac; MEL-B: R_1_ = Ac, R_2_ = H; MEL-C: R_1_ = H, R_2_ = Ac, n = 6–10; MEL-D: R_1_ = R_2_ = H, n = 4–14.

Betulinic acid (BA), also known as 3β-Hydroxy-lup-20(29)-en-28-oic acid shown in Figure 1A, is chemically a lupan-skeleton pentacyclic triterpene widely distributed in plants [6], and exhibits a variety of biological properties, such as its antiviral activities (especially against HIV) [7], its anti-inflammatory and cytotoxic effects [8]. Since BA was first discovered to have a strong selective inhibitory ability against human melanoma cells by Pisha et al. in 1995 [9], it has been widely reported to have great potential in cancer treatment due to its antitumor activities. According to the literature, BA has achieved remarkable anti-tumor impacts on melanoma cells and several types of solid tumors, including lung cancer, colorectal, glioblastoma, breast cancers, and prostate cancer [10]. Though the types of tumor cells are much different, the mechanism seems to be the same that both the stimulation of cell apoptosis and the inhibition of kinases play a leading role [11]. Recently, human liver cancer was generally studied and its various anti-cancer compounds were reported. It is worth noting that BA can inhibit the proliferation of human liver cancer HepG2 cells by influencing its cell cycle and mitochondrial membrane potential [12].

In recent years, there has been more attention paid to glycolipid biosurfactants (BSs), which play an important role in liposomes, due to its remarkable drug-carrying function and stabilizing effect [13]. Mannosylerythritol lipid-A (MEL-A), as shown in Figure 1B, has been reported to significantly increase the transfection efficiency of liposomes and accelerate the fusion of liposome-DNA complex and membrane to promote its entry to cell [14]. In addition, MEL-A exhibits marked antitumor activities against cancer cells, especially myelogenous leukemia K562 cells, promyelocytic leukemia HL60 cells, basophilic leukocyte KU812 cells and mouse melanoma B16 cells [15]. Presently, cancer has posed a threat to public health and been a major cause of human death worldwide. As the sixth most common cancer, it has been reported that there are 598,000 deaths annually caused by Hepatocellular carcinoma (HCC) out of 626,000 cases worldwide, which has been a major public health threat in the world [16]. Due to the potent toxic adverse effects of proposed drugs, there is an urgent demand to exploit novel substance to combat this devastating disease. Currently, no relevant studies are reported on the promotion of hydrophobic drug delivery by MEL-A modified liposomes and the enhancement of anticancer activity of liposomes. Therefore, the aim of this study is to investigate the enhancement of MEL-A on the inhibition of HepG2 cell proliferation, promotion of cell apoptosis, cell cycle block and mitochondrial membrane potential changes in BA-loaded liposomes, in order to provide the basis of further development and use of MEL-A.

## 2. Results

### 2.1. The Effect of Ratio of SPC to Chol on the Size of Liposomes

**Figure 2:**
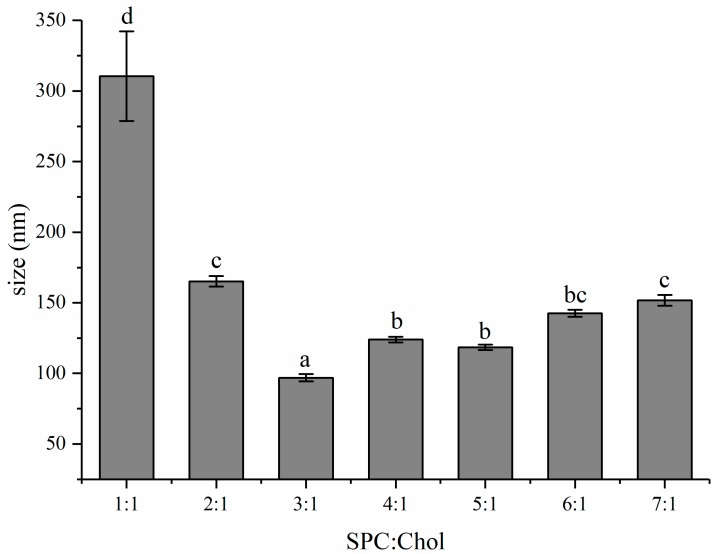
The effects of soy phosphatidylcholine-cholesterol ratio on the size of liposomes. Different letters in this figure represent significant difference among different treatment (*p* < 0.05). Error bars represent standard deviations (SD) (*n* = 3).

The ratio of SPC to Chol has significant effect on the particle size of liposomes as shown in Figure 2. It was obviously observed that the size of liposomes was 80.90 ± 0.50 nm at the ratio of 3:1, which is smallest among all investigated groups. Moreover, with increasing mass ratio of Chol, liposome size first decreased, reaching the small point at an SPC:Chol mass ratio of 3:1, before increasing again upon further addition of Chol. Cholesterol not only has influence on liposome size by incorporating into the lipid bilayers, but also regulates the mobility, permeability and stability of liposomes [17]. Based on the size distribution data, the determined ratio of 3:1 was chosen to conduct the following experiments.

### 2.2. The Effect of MEL-A Adding Ratio on the Size, PDI and Zeta Potential of Liposomes

**Figure 3:**
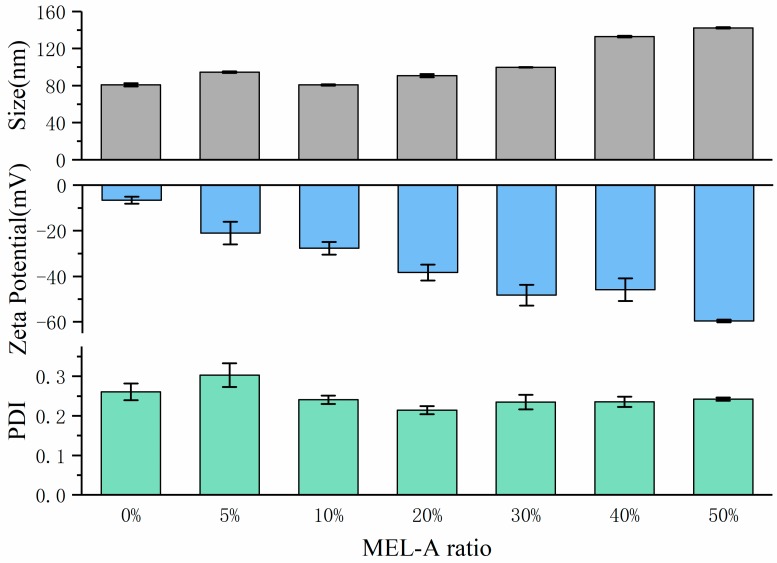
The effect of MEL-A adding ratio on the size, PDI and zeta potential of liposomes. SPC:CHOL was determined as 3:1. Error bars represent standard deviations (SD) (*n* = 3).

The influence of MEL-A addition ratio on size and zeta potential of liposomes has been exhibited in Figure 3. In the figure, the liposome particle size was 80.70 ± 1.67 nm without MEL-A added. When adding 5% MEL-A, the liposome size increased to 94.60 ± 0.85 nm, which may be due to the incorporation of MEL-A into the liposome bilayer. Nevertheless, the liposome size was reduced to 80.70 ± 0.78 nm along with MEL-A ratio further increasing to 10%. Thereafter, the particle size was in positive relation to the adding ratio of MEL-A. Meanwhile, after adding above 10% MEL-A, the PDI value of the liposome decreased to 0.25, which indicates that the addition of MEL-A helps to form a liposome system with more uniform size distribution. The size distribution of each group of liposomes is presented in Figure S1. Moreover, after the presence of MEL-A, the absolute value of the zeta potential of the liposome system increased significantly (actual value was negative).

### 2.3. The Effect of Loaded BA on Size, PDI and Zeta Potential of Liposomes

**Figure 4:**
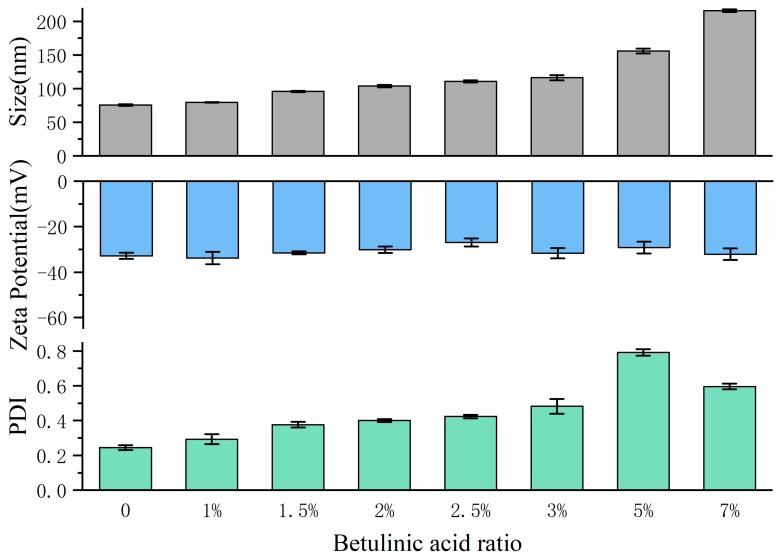
The effect of adding BA on the size, PDI and zeta potential of liposomes. MEL-A ratio was set as 5% in this investigation, SPC:CHOL was determined as 3:1. Error bars represent standard deviations (SD) (*n* = 3).

The effect of loaded BA on the size, PDI and zeta potential of liposomes was investigated after confirming the suitable MEL-A concentration and ratio of SPC: Chol was 10% and 3:1, and the data were presented in Figure 4 and Figure S2. With the increase of loaded BA ratio, the particle size of liposomes gradually increased and the PDI value also showed an upward trend. The particle size distribution of liposomes gradually transformed from a relatively uniform single peak to a bimodal and multi-peak system. On the other hand, the addition of BA had little effect on the zeta potential of liposomes, and the value of each group was basically maintained at about −30 mV. Considering the particle size, particle size distribution and BA package load, the optimal amount of BA was determined as 1% for the following experiments.

### 2.4. Changes in Particle size, PDI, Zeta Potential and Encapsulation Efficiency of BA-Loaded Liposomes

After optimizing the ratio of each component of the BA-loaded liposome, SPC-Chol liposomes (SC), SPC-Chol-BA liposomes (SCB), SPC-Chol-MEL-A liposomes (SCM), SPC-Chol-MEL-A-BA liposomes (SCMB) were prepared according to the optimal conditions, and the particle size, PDI and zeta of the samples were determined by laser nanometer. All the results as well as encapsulation efficiency are presented in Table 1. The data of particle size and zeta potential suggested that liposomes modified by MEL-A could reduce the increase in particle size, and improve the stability of liposomes. Nevertheless, the encapsulation efficiency (EE) of BA in SCMB was little lower than in SCB. This data implied that the modification of MEL-A had insignificant effect on the EE of BA. Referred to the reported literature, the modification of surfactant may have influence on the phospholipid bilayer structure of liposomes, and also improve the membrane permeability [9], which is the reason for the effect on BA’s encapsulation efficiency. As we know, MEL-A is a kind of biosurfactants and may play the same role in this study.

### 2.5. Morphology Observation of Different Liposomes

**Figure 5:**
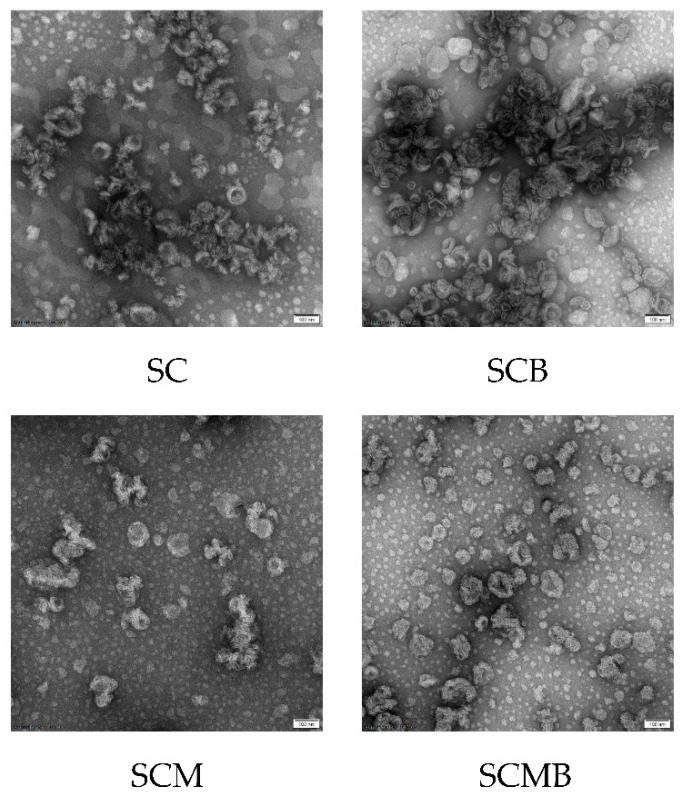
The TEM observation of SC, SCB, SCM and SCMB liposomes (The scales are 100 nm).

The morphology of liposome structure was observed by transmission electron microscope (TEM), as exhibited in Figure 5. In the images of TEM, liposome particles with bulbous and crumpled appearance, like the structure of erythrocyte, were clearly observed, which is consistent with results reported in the previous report [18]. Besides, the particle size of the liposome system is basically around 100 nm, which is in accordance with that measured by dynamic light scattering (DLS). Finally, the data of TEM showed that several liposome systems have been synthesized successfully and the particle size was about 100 nm.

### 2.6. Anti-Tumor Activity of Liposomes in HepG2

#### 2.6.1. Inhibitory Effect on HepG2 Cells Growth

**Figure 6:**
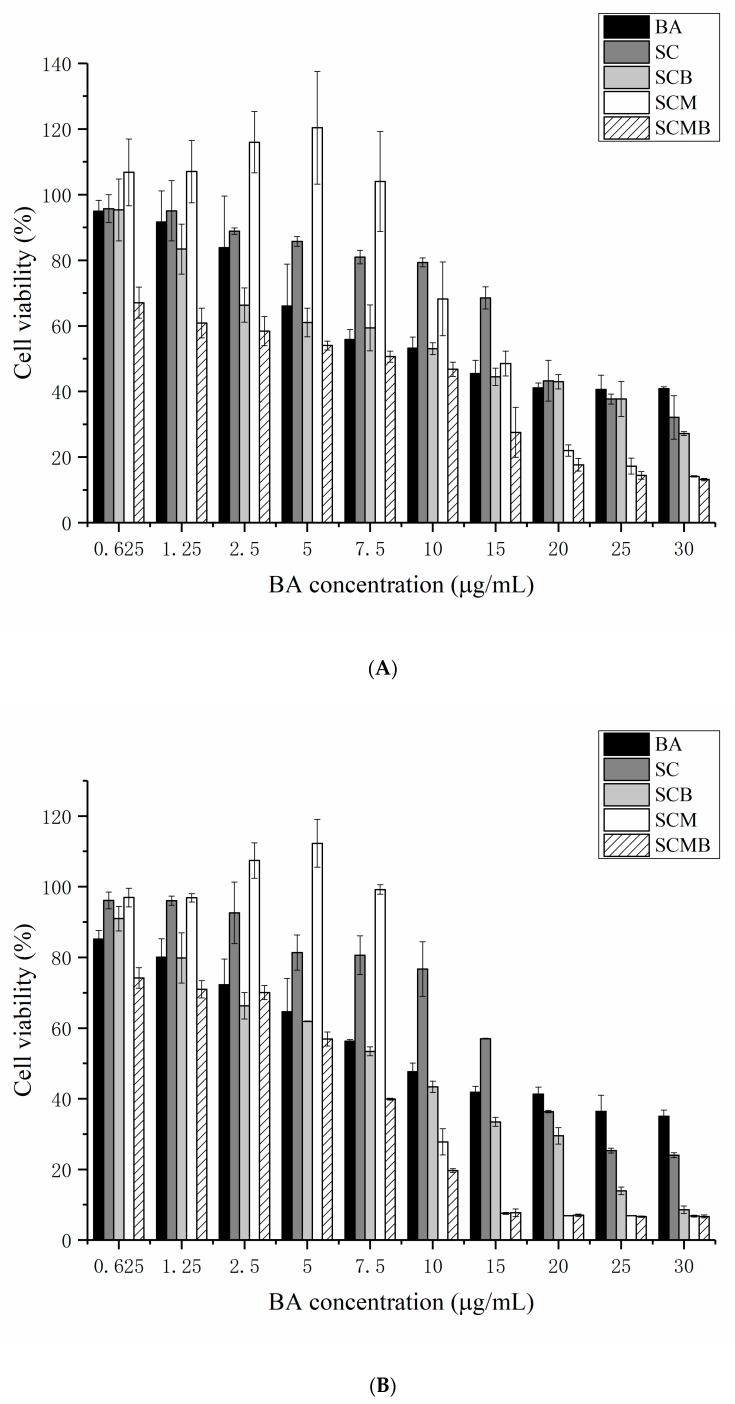
The proliferation changes of HepG2 cells under different treatments for 24 h (**A**) and 48 h (**B**). At each concentration of BA, SC and SCM are prepared under the same condition as well as SCB and SCMB whether adding BA or not.

The MTT method was used to determine the growth inhibition of HepG2 cells in human liver cancer by BA and liposomes containing different concentrations of BA, as shown in Figure 6. It can be seen that BA, SC, SCB and SCMB showed an obvious dose-effect relationship on the growth inhibition of HepG2 cells, and the cell survival rate gradually decreased with the increase of BA concentration. By comparing Figure 6A with Figure 6B, it is apparent that the growth inhibition of samples increased with the extension of treatment time. Based on the MTT data, the IC_50_ value of each sample can be calculated: the IC_50_ values of BA, SC, SCB, SCM and SCMB for 24 h were 13.24, 22.39, 10.84, 8.52 and 3.42 g/mL, respectively. The IC_50_ values for 48 h were 10.53, 15.81, 6.02, 7.45 and 3.11 g/mL, respectively. As a classical drug carrier, liposomes do not only enhance the solubility of drugs, but also improve the affinity of compounds to cancer cells and enhance the penetration of compounds to cancer cells, so as to improve the bioavailability of drugs owing to that liposomes may act as permeation enhancers by penetration of individual lipid components. In this work, IC_50_ values of SCB and SCMB are lower than that of BA, indicating that BA-containing liposomes are more potent in inhibiting HepG2 cells growth.

#### 2.6.2. Effect on Cell Apoptosis of HepG2

**Figure 7:**
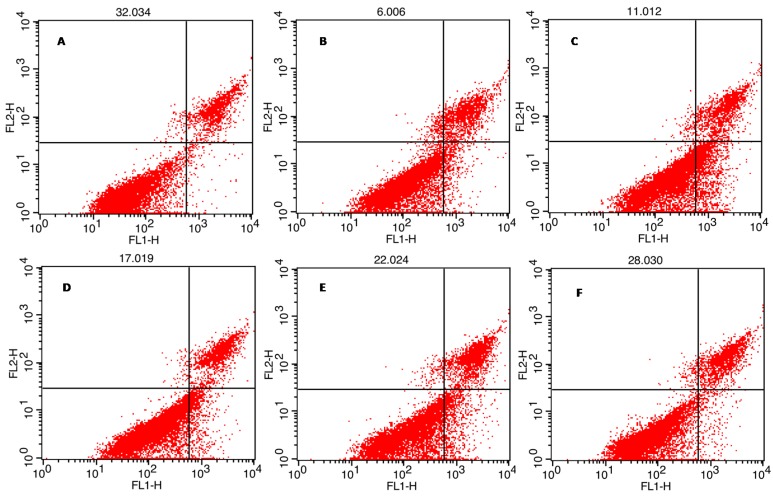
Effect of different treatments on the apoptosis of HepG2 cells. (**A**) Blank; (**B**) BA; (**C**) SCMB; (**D**) SCB; (**E**) SCM; (**F**) SC. FL1-H is the tunnel to collect Annexin V-FITC signal and FL2-H is the tunnel to collect PI signal.

Apoptosis refers to the independent and orderly programmed cell death controlled by genes to maintain the stability of internal environment, involving the activation, expression and regulation of a series of genes. Flow cytometer was usually used to further confirm the promoting role of BA-carrying liposomes containing MEL-A in HepG2 cell apoptosis. After being treated for 48 h, cells were stained by Annexin V-FITC and PI, and then detected by flow cytometer, as shown in Figure 7 and Table 2. Presently, the literature has revealed that BA can enhance the early apoptosis rate of HepG2 cells [12]. Compared with the control, the present results showed that the experimental group treated with liposomes loading BA could increase the proportion of early apoptosis cells and total apoptosis cells significantly, which is in accordance with the previous studies. Moreover, the inducing apoptosis effects of SCMB and SCB are both higher than that of BA alone. Meantime, MEL-A-modified liposomes (SCMB) revealed more pronounced effect on HepG2 cells than without MEL-A modification (SCB) (Table 2). Therefore, these results have proved that the anticancer effect of BA can be enhanced greatly via BA-loaded liposome system.

#### 2.6.3. Effect on Cell Cycle of HepG2

**Figure 8:**
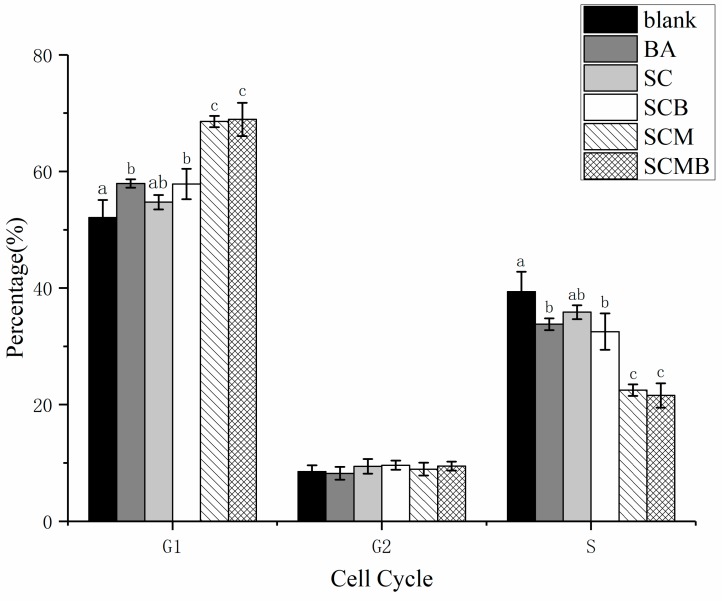
Comparative analysis of cell cycle distribution under different treatments. Different letters in the figure represent significant difference among different treatments (*p* < 0.05).

The influence of different BA-treating groups on cell cycle of HepG2 is also presented in Figure 8. It is apparent that the percentage of cells in G1 phase increased and there was no difference of cells in G2 phase after treated with BA, while the proportion of cells in S phase decreased significantly. In the G1 phase, RNA and protein synthesis is mainly carried out, and DNA synthesis is prepared for entering S phase. BA and MEL-A may affect and interfere with the RNA and protein preparation of cells in G1, so that cells cannot enter S phase. G2 phase is the late stage of DNA synthesis and the preparation period for mitosis. During this period, DNA synthesis was terminated, and a large amount of RNA and proteins were synthesized. Compared to G1 and S phase, there is no difference in G2 phase, which means that the treatment of BA and liposomes have little influence on G2 phase cells.

#### 2.6.4. Effect on Mitochondrial Membrane Potential

**Figure 9:**
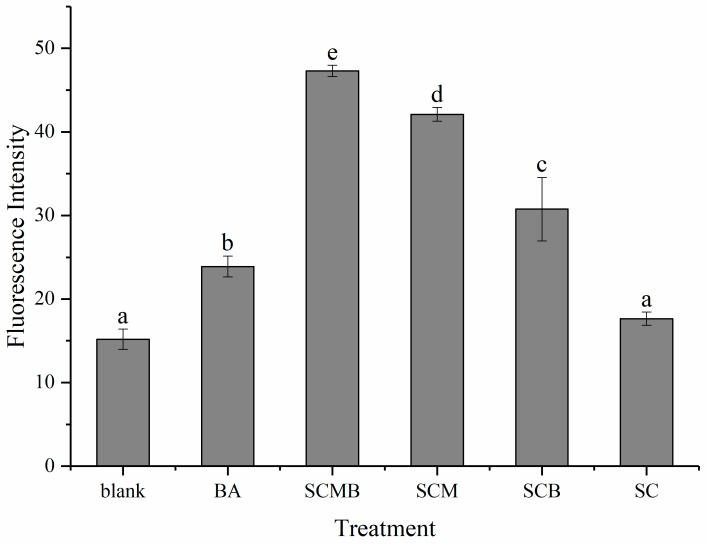
Mitochondrial membrane potential in HepG2 cells by flow cytometry under different treatments. Using different letters in the figure represents significant difference among six different treatments (*p* < 0.05).

Rhodamine 123 is a mitochondrial transmembrane potential indicator that can penetrate cell membranes, which can enter the mitochondria in normal cells, resulting in the disappearance or lower fluorescence intensity. When the cell apoptosis occurs, the integrity of the mitochondrial membrane is destroyed, and the mitochondrial membrane potential collapses, leading to the leakage of Rhodamine 123 as well as the increase of the fluorescence intensity. Therefore, there is a negative correlation between mitochondrial membrane potential and fluorescence intensity. Flow cytometry was applied to detect the influence of different drug treatments on mitochondrial membrane potential of HepG2 cells, and the results were presented in Figure 9 and Figure S3. There is insignificant difference between SC and the blank group, suggesting SC has a little impact on mitochondrial membrane potential. However, the fluorescence intensity of BA group was significantly higher than that of the blank group, implying that BA-inducing HepG2 apoptosis was likely related to the mitochondrial pathway.

## 3. Discussion

This study has successfully constructed soy phosphatidylcholine-cholesterol liposomes modified by MEL-A and loading betulinic acid, and obtained the optimal liposome formulation with 3:1 of SPC to Chol, 10% MEL-A and 1% BA. With reducing the Chol concentration, the particle size of liposomes has shown the phenomenon of falling and then rising, which meant that the ratio of SPC to Chol has a significant influence on the size of liposomes. However, the best formulation of liposomes still remained further optimization with response surface methodology in the future research. Besides, previous literature has discovered that an appropriate proportion of cholesterol can enhance the density and rigidity of liposomes to prevent drug leakage; however, the lipid bilayers is easily damaged when the content of cholesterol is too high [19]. Meanwhile, the addition of MEL-A affected the particle size, PDI and zeta potential as well and resulted in a more uniform particle size distribution and improved stability. Actually, zeta potential reflects the stability of the liposome: the higher absolute value means the greater surface charge intensity of the liposome and the stronger mutual repulsion [20]. Therefore, the increase in the absolute value of zeta potential after the addition of MEL-A indicates that MEL-A dramatically improved the stability of the liposome. In particular, the negative charge on the surface can reduce the absorption of non-specific proteins in the circulation of liposomes [21,22,23] and avoid the rapid elimination of liposomes by the reticuloendothelial system in vivo [24]. However, excessive positive or negative potentials of liposomes are also detrimental to the decrease of adsorption between liposomes, liposomes and other surfaces [25].

Encapsulating BA will remarkably increase the size of liposomes, while excessive BA will degrade the size distribution of liposomes. As a lipophilic pentacyclic triterpene, BA is mainly embedded in the phospholipid bilayer of liposomes when loaded in the liposomes. Therefore, the loading of BA would change the particle size and distribution of liposomes. Remarkably, BA, SC, SCB, SCM and SCMB have excellent inhibitory activity against the proliferation of HepG2 cells with a dose-effect relation. In addition, the BA-encapsulated liposomes, modified by MEL-A, had the lowest IC_50_ value, which means that the modification of MEL-A can enhance the inhibition effect of BA-loading liposomes against cancer cell growth. Interestingly, MEL-A liposomes without BA also have smaller IC_50_ values. It was supposed that there may be two reasons for this phenomenon: Firstly, MEL-A can improve the affinity of liposomes to cells, and significantly increase the efficiency of gene transfection of liposome-DNA complexes by promoting membrane fusion [26,27]. Secondly, the ability to inhibit the growth of cancer cell by MEL-A itself is also an important cause. It has been reported that MEL-A could inhibit growth and promotes cell differentiation in melanoma B16 cells and leukemia HL60 and K562 cells [28]. However, the survival rate of HepG2 cells increased to some extent at low concentration of SCM, due to the inhibitory activity of MEL-A is weak at a low concentration in the liposome system, and its surface property facilitates the uptake of nutrients by cancer cells, the specific reason of which still remains to be studied. It is known that cell apoptosis could be activated by all kinds of damage in or out of the cell, resulting a programmed death of cell, thus inducing apoptosis has become the common way for anticancer compounds to treat tumor cells [29]. In a previous study, betulinic acid (BA) has gained a huge significance in the recent years for its strong cytotoxicity, while the normal cells and tissues are rarely affected by BA [30]. In our study, the experimental group treated with BA or liposomes could increase the proportion of early apoptosis cells significantly. Meanwhile, the impact of BA-carrying liposomes is stronger than BA alone, and MEL-A-modified liposomes are more effective than ordinary liposomes. These results further verify the enhanced ability of liposome entrapment to exert drug effects, and also demonstrate that the modification of liposomes by MEL-A improves the anticancer efficacy of BA-loaded liposomes.

Generally, the cell cycle of normal cells keeps regular, while the disturbance of cell cycle is often regarded as a hallmark of tumor cells. Therefore, there is a way of anti-cancer drugs to induce apoptosis by arresting the cell cycle at the G1, S, or G2/M phase [31]. In this study, BA treatment caused G1 block of cells, preventing the G1 phase cells from entering the S phase. In particular, SCMB not only had a more obvious effect than BA, but also had a significantly stronger effect of cell cycle block than SCB. In addition, the block effect of cell cycle of SCM was apparently stronger than other groups except SCMB, which means MEL-A may not only enhance the drug use of BA in liposomes, but also play an anticancer role itself. In fact, the synthesis of RNA and protein are mainly carried out in the G1 phase, preparing for DNA synthesis in the S phase. As a result, BA and MEL-A may affect and interfere with the RNA and protein preparation of the cells in G1, so that the cells cannot enter S phase. Recent studies have shown that there is close relation between mitochondria and apoptotic processes, and the decline or even collapse of mitochondrial membrane potential is considered to be one of the early features of cell apoptosis. Once the mitochondrial membrane potential collapses, cell apoptosis is irreversible [32]. From the data of Figure 6, there is an obvious upgrade of fluorescence intensity after adding BA into liposomes, which indicates that BA-inducing HepG2 cell apoptosis was related to mitochondrial pathway. Furthermore, MEL-A modified liposome can strengthen the destruction of mitochondrial membrane potential in HepG2 cells by BA. BA can induce the loss of mitochondrial membrane potential in cell-free mitochondrial systems, and this process can be inhibited by bongkrekic acid, an inhibitor of mitochondrial permeability transfer hole complex [32]. Therefore, the reason for the influence of BA on mitochondrial membrane potential may ascribe the action on mitochondrial permeability transfer hole. Besides, the fluorescence intensity of SCB was prominently higher than that of BA, further proving that liposomes can enhance the use rate of BA. In particular, the fluorescence intensity of SCMB and SCM group was significantly higher than that of other groups, indicating that MEL-A may play an important role in changing cell membrane permeability and promoting the infiltration of BA into targeted cells. Future study should focus on the in vivo evaluation of this developed liposomes with anti-cancer activity.

The finding in this work indicates that MEL-A modification can improve the penetration and delivery of liposome BA to HepG2 cells, and synergistically enhance the anticancer activity of liposomes, which provides a basis for the further development of MEL-A application in anticancer liposomes and an interesting research field for the employment of BA in vivo.

## 4. Materials and Methods

### 4.1. Chemicals and Reagents

MEL-A were produced by *Pseudozyma aphidis* DSM70725 in our laboratory. According to Fan et al., MEL-A was purified from the crude MELs using silica gel column and confirmed by TLC (Silica gel GF254, Pibo Biotechnology Co., Ltd., Hangzhou, China), trichloromethane:methanol:water = 65:15:2, *v*/*v*), which is up to 90% purity [28]. The soy lecithin (>98%) was purchased from Shanghai Aladdin Biochemical Technology Co., Ltd. (Shanghai, China). The betulinic acid (97%) was purchased from Development Co., Ltd. (Shanghai, China). All the chemicals used in this study were of analytical grade.

### 4.2. Preparation of Liposomes

#### 4.2.1. Optimization of Soybean Lecithin and Cholesterol Mass Ratio

Soy phosphatidylcholine (SPC)-cholesterol (Chol) liposomes were prepared by thin film hydration method. Different weight of SPC and Chol were mixed and dissolved in chloroform, according to the mass ratio of SPC to Chol (1:1, 2:1, 3:1, 4:1, 5:1, 6:1, 7:1, as the molar ratio of 1.19:1, 2.37:1, 3.57:1, 4.76:1, 5.95:1, 7.14:1, 8.33:1). Then the mixture was evaporated at 40 °C and 150 rpm and allowed to stand at 4 °C overnight. The final lipid film was obtained thoroughly purged with nitrogen in order to remove the residual organic reagent. Finally, the lipid film was dispersed into the phosphate saline buffer (PBS) using an ultrasonic cleaner (JP-040S, Skymen Cleaning Equipment Shenzhen Co., Ltd., China) for 15 min, and sonicated for 3 min using an ultrasonic cell grinder (JY96-IIN, Ningbo Xinzhi Biotechnology Co., Ltd. China). The size of the liposome was determined by a Zetasizer Nano ZS90 (Malvern Instruments Ltd., Malvern, Worcestershire, UK) at a wavelength of 633 nm.

#### 4.2.2. Optimization Determination of MEL-A Adding Ratio

To determine the optimal addition ratio of MEL-A, different masses of MEL-A, accounting for 0%, 10%, 20%, 30%, 40% and 50% of the total lipid, mixed with SPC and Chol at the optimal ratio and dissolved in chloroform. Then the thin film was acquired and carried out as the same as indicated before. At last, the particle size and polydispersity index (PDI) of the liposome were measured as referred to 4.3.1 to confirm the optimal addition ratio of MEL-A.

#### 4.2.3. Optimization of Betulinic Acid Loaded Concentration

The optimal ratio of betulinic acid (BA) in the liposome was also investigated. SPC, Chol and MEL-A were dissolved in chloroform according to the optimal proportion, and BA at different concentrations (0%, 1%, 1.5%, 2%, 2.5%, 3%, 5% and 7% of the total lipid) was dissolved in chloroform and mixed well with the lipid solution. Then, the thin film was acquired and conducted as the same as indicated before. Lastly, the particle size and PDI of the liposomes were measured to confirm the optimal addition concentration of BA.

### 4.3. Characterization of Liposomes Modified with MEL-A and Loading Betulinic Acid

#### 4.3.1. Measurement of Size Distribution and Zeta Potential

Liposomes were prepared at 3.75 mg/mL concentration for the detection of size distribution, PDI and zeta potential using a Zetasizer Nano ZS90 (Malvern Instruments Ltd., Malvern, Worcestershire, UK) by dynamic light scattering (DLS) at a wavelength of 633 nm., under the condition that the temperature was set at 25 °C and the measurement angle was 90°.

#### 4.3.2. Morphology Observation

The morphology of the liposomes was observed using JEM-1200EX transmission electron microscope (JEOL, Tokyo, Japan). After the sample was adsorbed on the surface of the copper mesh, it was stained with uranyl acetate, and the sample was observed after stabilization.

#### 4.3.3. Measurements of Encapsulation Efficiency

The encapsulation efficiency of liposome containing betulinic acid was determined by ultra-high speed-low speed centrifugation method. The liposomal system was centrifuged at 1000 rpm for 10 min to remove free betulinic acid. The supernatant was centrifuged at 12,000 rpm for 20 min, and the liposome precipitate was combined with methanol and chloroform (1:1) to destroy the structure. The content of betulin in the liposomes was determined referring to the method of Wu et al. [33] as detection wavelength 210 nm, Reverse C18 Column (250 mm × 4.6 mm id, 4 μm; Waters), mobile phase acetonitrile: water = 91:9, flow rate 1.0 mL/min under 30 °C. Encapsulation efficiency of the liposomes was calculated by the following formula:

EE (%) = (encapsulated betulinic acid)/(encapsulated betulinic acid+unencapsulated betulinic acid) × 100%

### 4.4. Culture of HepG2 Cells

In this study, HepG2 human hepatoma cells were purchased from Shanghai Institute of Biochemistry and Cell Biology, CAS (Shanghai, China). The cells were cultured in dulbecco’s modified eagle medium (DMEM) supplemented with 10% (*v*/*v*) heat-inactivated fetal bovine serum and penicillin/streptomycin (1 × 10^5^ U/L) and maintained at 37 °C in a 5% CO_2_ complete humidified incubator [34]. The cells were sub cultured when growing to the logarithmic phase.

### 4.5. Cell Viability Assay with MTT

The MTT test is a classic method to evaluate cell viability in vitro and the results are in positive relation to the number of viable cultured cells [35]. SPC-Chol liposomes (SC), SPC-Chol-BA liposomes (SCB), SPC-Chol-MEL-A liposomes (SCM), SPC-Chol-MEL-A-BA liposomes (SCMB) were prepared and dispersed in the medium, and the solution was filtered through 0.2 μm membrane. The total lipid concentration of all liposomes is 125 times that of BA, so each concentration of BA represent one kind of preparing condition involving addition of other lipids. Specifically, BA was dissolved in water with dimethyl sulfoxide (DMSO) and diluted to a serial of concentrations (0.625, 1.25, 2.5, 5, 7.5, 10, 15, 20, 25 and 30 μg/mL) by gradient dilution method, and then added to form drug-loaded liposomes (SCB and SCMB). Meanwhile, other preparing conditions of SC and SCM keep the same with SCB and SCMB at different concentrations of BA. 100 μL of logarithmic phase cells (1.5 × 10^5^ cells/mL) were inoculated in every well of 96-well plates and incubated at 37 °C for 24 h. After cell attachment, the primary medium was removed and fresh mediums with different concentrations of BA, SC, SCM, SCB and SCMB were placed into each well, while every experimental group was repeated 6 times. The control group was set as adding only culture medium into the well. After incubating 24 h or 48 h, the medium was discarded and 20 μL of MTT reagent (5 mg/mL) was added and incubated at 37 °C for 3 h. Then, 150 μL of DMSO was added and shaken for 10 min. Finally, cell viability was measured at 570 nm and rectified at 630 nm. Following equation was used to calculate the inhibition of liposomes on HepG2 cells:

Cell viability = (OD_570_ nm (experimental group) − OD_630_ nm (experimental group))/(OD_570_ nm (control group) − OD_630_ nm (control group))

### 4.6. Cell Apoptosis Determination by Flow Cytometry

Cell apoptosis was detected by Annexin V-FITC/propidium iodide (PI) staining assay using flow cytometry [36]. To analyze liposomes induced apoptosis of HepG2 cells, logarithmic phase cell concentration was adjusted to 1.5 × 105 cells/mL and inoculated in 6-well plates. Cells were harvested after 24 h treatment with 5 μg/mL BA or different liposomes suspensions and digested trypsin. The test method was performed according to the Annexin V-FITC/PI Apoptosis Detection Kit instructions (Lianke Biotechnology Corporate Ltd. Hangzhou, China). At the end of the staining process, the stained cells were analyzed with flow cytometry (FACSCaLIBUR, Becton, Dickinson and Company, Newark, NJ, USA).

### 4.7. Cell Cycle Analysis by Flow Cytometry

For cell cycle analysis, logarithmic phase cell concentration was adjusted to 1.5 × 10^5^ cells/mL and seeded in 6-well plates. After culturing for 24 h, cells were treated with 10 μg/mL BA or liposome suspensions for 24 h and then digested with trypsin. Then the cells were collected by centrifugation at 1500 rpm at 4 °C and washed by PBS twice, and the supernatant was discarded. Cells were then resuspended in pre-cooled ethanol at −20 °C overnight [37]. Cells were washed by PBS twice before detection and then resuspended in DNA staining solution (PI) (MultiSciences (Lianke) Biotech Co., Ltd.), and cultured in the dark at room temperature for 30 min. The stained cells were measured by flow cytometry to analyze the cell cycle.

### 4.8. Mitochondrial Membrane Potential Assessment

Mitochondria is capable of controlling the level of released proapoptotic proteins to promote apoptotic cell death and also play significant roles in non-apoptotic cell death [38]. Mitochondrial membrane potential (MMP) was detected by the Rhodamine 123 staining. Briefly, logarithmic phase cell concentration was adjusted to 1.5 × 10^5^ cells/mL and seeded in 6-well plates. After culturing for 24 h, cells were treated with 10 μg/mL BA or liposome suspensions for 24 h and then digested with trypsin. Then the cells were collected by centrifugation at 1500 rpm at 4 °C and washed by PBS twice, and the supernatant was discarded. Subsequently, HepG2 cells were resuspended in PBS adding 10 μL Rhodamine 123 dye and incubated at 37 °C for 10 min. Finally, fluorescence intensity of cells was assessed by flow cytometry, which the excitation wavelength was 488 nm while the detection wavelengths were set at 530 nm.

### 4.9. Statistical Analysis

All experiments were carried out in triplicate. Differences between two samples were evaluated by a Student’s *t*-test and considered to be significant at *p* < 0.05 in this study.

## 5. Conclusions

This study has constructed soy phosphatidylcholine-cholesterol liposomes modified by MEL-A and loading betulinic acid, and obtained the liposome formulation with 3:1 of SPC to Chol, 10% MEL-A and 1% BA. The particle size of liposomes was significantly influenced by ratio of SPC to Chol, and decreased first and then increased with reducing the Chol concentration. The incorporation of MEL-A affected the particle size, PDI and zeta potential as well and resulted in a more uniform particle size distribution and improved stability. Moreover, BA, SC, SCB, SCM and SCMB have marked inhibitory impact against the proliferation of HepG2 cells with a dose-effect relation. In addition, the modification of MEL-A in liposomes can significantly enhance cell apoptosis (early apoptosis) and G1 phase block of cell cycle, which were both induced by BA. In particular, MEL-A modified liposome can strengthen the destruction of mitochondrial membrane potential in HepG2 cells upon BA treatment. The finding indicates that MEL-A modification can improve the penetration and delivery of liposome drugs to cells, and synergistically enhance the anticancer activity of BA-carrying liposomes, which provides a basis for the further development of MEL-A application in anticancer liposomes and a new research idea for the employment of BA in the treatment of liver cancer.

## Figures and Tables

**Table 1:** The size, PDI, zeta potential and EE of liposomes.

Group	Size (nm)	PDI	Zeta Potential (mV)	EE (%)
SC	75.96 ± 0.98 ^a^	0.344 ± 0.004 ^c^	−11.2 ± 0.361 ^a^	/
SCB	128.90 ± 0.26 ^d^	0.282 ± 0.008 ^b^	−10.9 ± 1.820 ^a^	82.21 ± 0.41 ^a^
SCM	81.00 ± 0.46 ^b^	0.221 ± 0.002 ^a^	−27.6 ± 3.410 ^b^	/
SCMB	89.83 ± 0.92 ^c^	0.348 ± 0.012 ^c^	−33.5 ± 2.390 ^c^	80.44 ± 1.19 ^a^

In this experiment, liposomes were constructed under 3:1 of SPC:CHOL, 10% MEL-A and 1% loading BA. Different superscripts in the column represent significant differences among different treatments (*p* < 0.05). / means without BA incorporation.

**Table 2:** The comparison of apoptosis rate under different treatments.

Treatment	Late Apoptosis (%)	Early Apoptosis (%)	Total Apoptosis (%)
Blank	14.75 ± 1.18 ^a^	1.36 ± 0.21 ^a^	16.11 ± 1.40 ^a^
BA	14.64 ± 2.25 ^a^	5.06 ± 0.45 ^b^	19.70 ± 2.06 ^b^
SCMB	16.31 ± 1.14 ^a^	15.36 ± 0.62 ^e^	31.67 ± 0.56 ^d^
SCB	16.27 ± 0.78 ^a^	9.28 ± 0.68 ^d^	25.55 ± 1.12 ^c^
SCM	14.24 ± 1.23 ^a^	6.42 ± 0.50 ^c^	20.66 ± 1.61 ^b^
SC	16.30 ± 1.35 ^a^	1.93 ± 0.52 ^a^	18.23 ± 1.88 ^ab^

## Supplementary Materials

The following are available online, Figure S1: The size distributions of liposomes with different concentrations of MEL-A, Figure S2: The effect of adding BA on the size distribution of liposomes, Figure S3: Mitochondrial membrane potential in HepG2 cells by flow cytometry under different treatments.

## Abbreviations

MEL-A	Mannosylerythritol Lipid-A
SPC	Soy Phosphatidylcholine
Chol	Cholesterol
BA	Betulinic Acid
PDI	Polydispersity Index
SC	Soy Phosphatidylcholine-Cholesterol Liposomes
SCB	Soy Phosphatidylcholine-Cholesterol-Betulinic Acid Liposomes
SCM	Soy Phosphatidylcholine-Cholesterol-Mannosylerythritol Lipid-A Liposomes
SCMB	Soy Phosphatidylcholine-Cholesterol-Mannosylerythritol Lipid-A-Betulinic Acid Liposomes
EE	Encapsulation Efficiency
TEM	Transmission Electron Microscope
