# Supplementary material for: Synthesis, Characterization of Liposomes Modified with Biosurfactant MEL-A Loading Betulinic Acid and Its Anticancer Effect in HepG2 Cell
Source: Molecules. 2019 Oct 31;24(21):3939. doi: 10.3390/molecules24213939 (PMC6864557; doi:10.3390/molecules24213939)
Supplement: Supplementary file 1 [file molecules-24-03939-s001.zip › molecules-622053-supplementary.docx]

**Supplementary files**

0%

10%

30%

50%

**Figure S1** The size distributions of liposomes with different concentrations of MEL-A.

| 0% | 1% |
| --- | --- |
| 1.5% | 2% |
| 2.5% | 3% |
| 5% | 7% |

Figure S2 The effect of adding BA on the size distribution of liposomes.


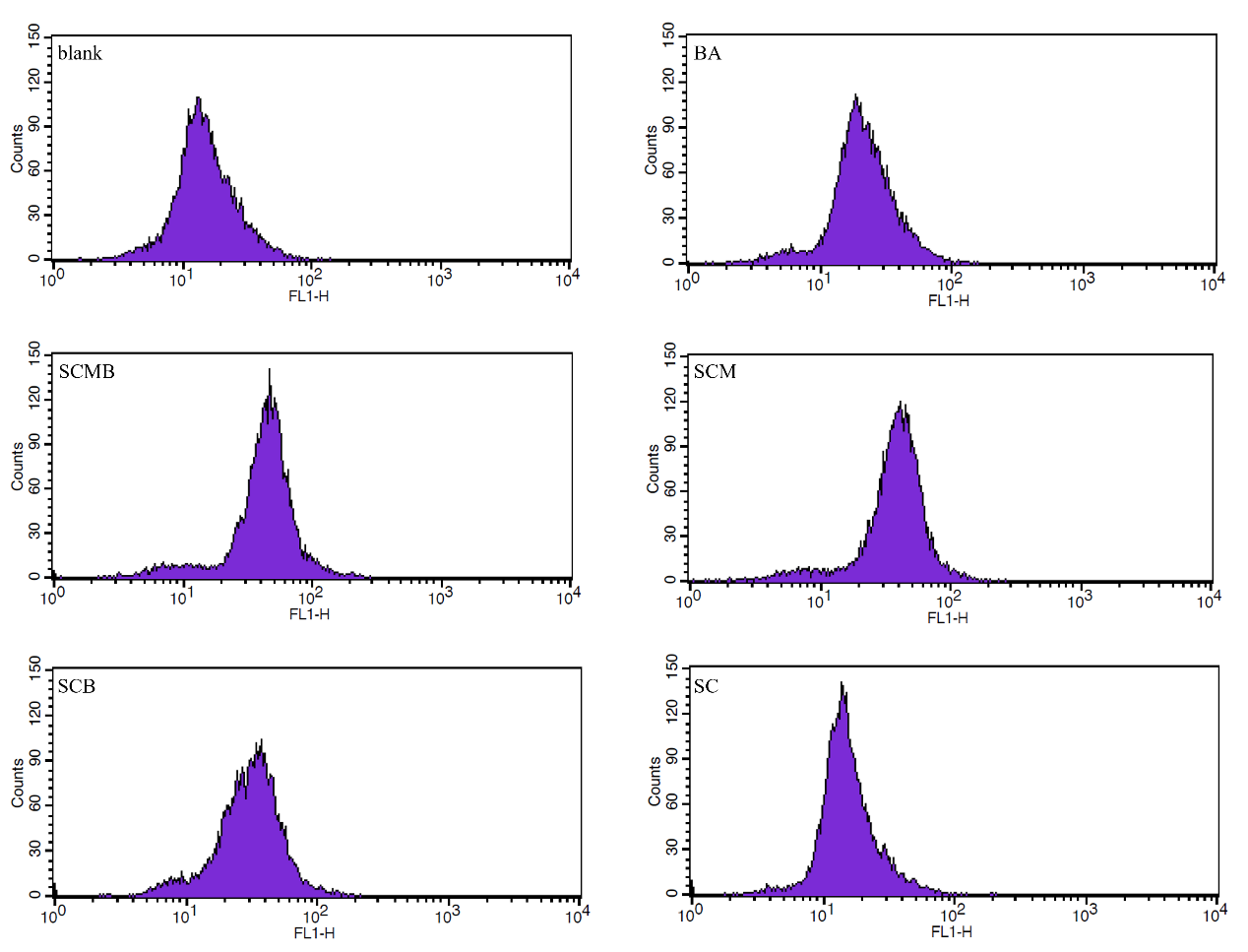


**Figure S3** Mitochondrial membrane potential in HepG2 cells by flow cytometry under different treatments.
